# Unlocking the potential of insect and ruminant host symbionts for recycling of lignocellulosic carbon with a biorefinery approach: a review

**DOI:** 10.1186/s12934-021-01597-0

**Published:** 2021-05-27

**Authors:** Gunasekaran Rajeswari, Samuel Jacob, Anuj Kumar Chandel, Vinod Kumar

**Affiliations:** 1 Department of Biotechnology, School of Bioengineering, College of Engineering and Technology, Faculty of Engineering and Technology, SRM Institute of Science and Technology, SRM Nagar, Chengalpattu Dist. , Kattankulathur, 603203 Tamil Nadu India; 2 Department of Biotechnology, Engineering School of Lorena (EEL), University of São Paulo, Lorena, 12.602.810 Brazil; 3 School of Water, Energy and Environment, Cranfield University, Cranfield, MK43 0AL UK

**Keywords:** Gut symbionts, Microbial community enrichment, Renewable carbon, Depolymerization, Recycling, Lignocellulolytic enzymes, Biofuels

## Abstract

Uprising fossil fuel depletion and deterioration of ecological reserves supply have led to the search for alternative renewable and sustainable energy sources and chemicals. Although first generation biorefinery is quite successful commercially in generating bulk of biofuels globally, the food versus fuel debate has necessitated the use of non-edible feedstocks, majorly waste biomass, for second generation production of biofuels and chemicals. A diverse class of microbes and enzymes are being exploited for biofuels production for a series of treatment process, however, the conversion efficiency of wide range of lignocellulosic biomass (LCB) and consolidated way of processing remains challenging. There were lot of research efforts in the past decade to scour for potential microbial candidate. In this context, evolution has developed the gut microbiota of several insects and ruminants that are potential LCB degraders host eco-system to overcome its host nutritional constraints, where LCB processed by microbiomes pretends to be a promising candidate. Synergistic microbial symbionts could make a significant contribution towards recycling the renewable carbon from distinctly abundant recalcitrant LCB. Several studies have assessed the bioprospection of innumerable gut symbionts and their lignocellulolytic enzymes for LCB degradation. Though, some reviews exist on molecular characterization of gut microbes, but none of them has enlightened the microbial community design coupled with various LCB valorization which intensifies the microbial diversity in biofuels application. This review provides a deep insight into the significant breakthroughs attained in enrichment strategy of gut microbial community and its molecular characterization techniques which aids in understanding the holistic microbial community dynamics. Special emphasis is placed on gut microbial role in LCB depolymerization strategies to lignocellulolytic enzymes production and its functional metagenomic data mining eventually generating the sugar platform for biofuels and renewable chemicals production.

## Introduction

**Fig. 1:**
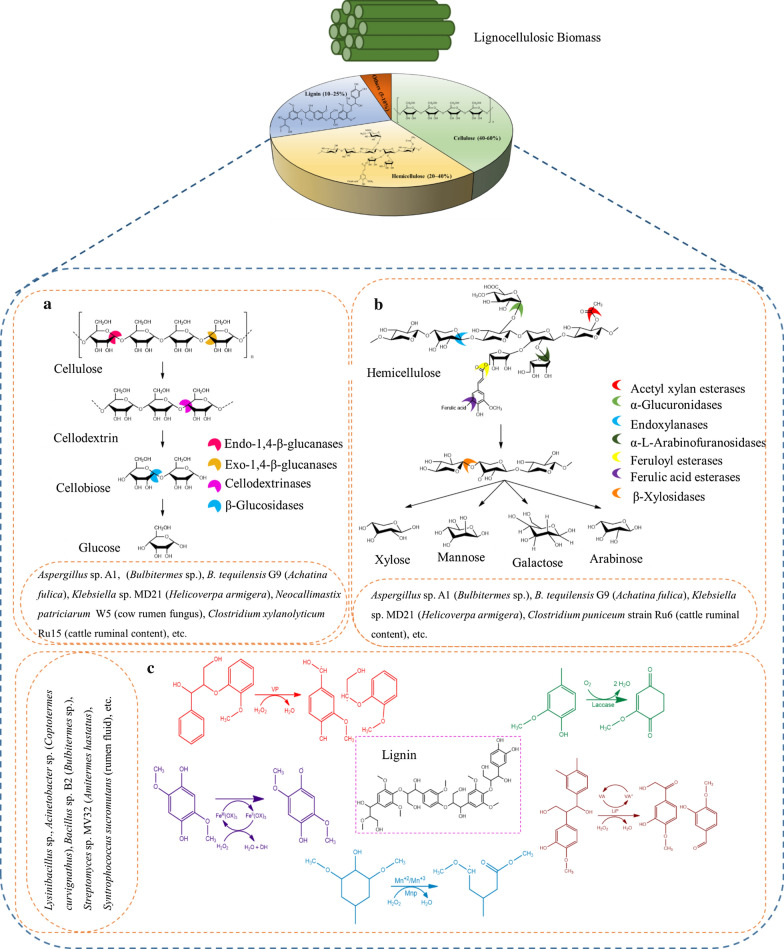
Schematic representation of structural composition of lignocellulosic biomass along with the action of major lignocellulolytic enzyme involved lignocellulosic biomass degradation. **a** Cellulase cocktails action mechanism; **b** Hemicellulase/xylanase action mechanism; **c** Ligninase/Laccase action mechanism. Adapted and modified from Xie et al. [10]; Sun et al. [11] and Janusz et al. [12]

In the field of renewable energy, bioconversion of lignocellulosic biomass (LCB) attempts to substantiate the earths carbon recycling by creating a green biorefinery approach. Production of renewable second generation fuels and value-added chemicals from lignocellulosic fractions leads to the establishment of sustainable green biorefinery. LCB, primarily composed of carbohydrate (cellulose—40%–60% and hemicellulose—20%–40%) and non-carbohydrate polymers (lignin—10%–25%) associated in a hetero-matrix form [1, 2]. The relative abundance of these polymers remains as a key factor to determine the optimal energy conversion route for several type of LCB. In biorefineries, LCB valorization involves the breakdown of carbohydrates and lignin into fermentable sugars and lignin monomers respectively via lignocellulolytic mechanism of innumerable enzymes or microbes from diverse genera and families [3]. Several lignocellulolytic enzymes involved in the complex LCB degradation includes ligninolytic oxidases (laccase and peroxidases- lignin peroxidase, manganese peroxidase, versatile peroxidase) and hydrolases (cellulases, hemicellulases/xylanase, amylases, pectinases, esterases, mannanases and chitinases) [4]. Figure 1 (a-c) highlights the mechanism of major lignocellulolytic enzymes that are involved in the LCB conversion. Based on the studies, lignin oxidization is typically initiated and facilitated by peroxidases superfamily using H_2_O_2_ as co-substrate, in turn the oxidation results in the free radicals formation along with reactive anions or cations [5, 6]. Laccase, a multicopper superoxidase family catalyze the oxidization of aromatic compounds and reduced phenols using O_2_ as cofactor [7]. In case of hydrolytic enzyme, microbial cocktail cellulase, hemicellulase and xylanase are generally possessed with the synergistic action as shown in the Fig. 1a and c where the product are monomeric sugars [8, 9].

Many fermented products obtained from the LCB processing necessitates an economical and ecological fermentative microbial strategy for its industrial feasibility in order to circumvent its recalcitrant nature. Evolution has developed the gut microbiome of several xylophagous insects and ruminants host eco-system in order to overcome its host nutritional constraints. Symbiotic gut microflora synergistically works as a carbon recycler by mineralization of plant biomass [13, 14]. Furthermore, in-depth discrimination of these distinct synergistic mechanism regarding the lignocellulose digestion strategy in gut microbiome is vital for biomimicry of wide range of LCB biodegradation platform. However, in context of growing bioeconomy, this natural gut microbiome necessitates an enrichment strategy i.e., in vivo/in vitro to attain an optimal desired microbial candidate thereby enhancing the functionality of microbial cell factories as lignocellulose recycler. Production of biofuels and various platform chemicals rely on systematic considerate of microbial metabolism within community. The extended investigation of this complex gut microflora facilitates the culture independent metagenomic approaches to evaluate the taxonomical and functional diversity of symbiotic gut microbial community. Thus, providing a comprehensive information about the microbial diversity, community dynamics and function of gut microbes is utmost important for the successful deployment of lignocellulose biorefineries.

The complexity of ruminants/insects gut microflora is of great concern as they are unculturable and then deciphering the novel Carbohydrate Active Enzyme (CAZymes) diversity encompassing an array of plant cell wall hydrolytic enzymes would involve functional metagenomics [15, 16]. Thus, mining of unexploited and unexplored oxidative/hydrolytic enzymes of gut microbial community lead to the bioprospecting of ideal biocatalysts of CAZymes family from the gut microbiome niche. This could ultimately contribute in the LCB degradation and further aids in the development of second generation biofuels and biochemicals sector. In this context, gut microbes possess diversity of LCB valorization techniques that includes pretreatment, enzymatic saccharolysis, fermentation and anaerobic digestion for its biorefinery based conversion process [17]. Recently, gut and rumen microbial isolates are being employed as consortia for the efficient cellulosic biofuel production. The cooperative microbial energetics is highly strengthened owing to its “division of labor” thus in turn, pave the way for imminent genetic, metabolic engineering and bioprospecting. This review article elucidates the microbial community enrichment, molecular tools on the horizon of microbial characterization, diverse perspective of LCB degradation mechanism and its conversion into second generation biofuels and renewable chemicals for unlocking the commercial latent of LCB based biorefinery.

## In vivo and in vitro techniques for gut microbial community enrichment

In the biorefinery framework unleashing the ability of gut and rumen microbial consortia towards conversion of LCB into biofuels and value-added products are gaining concern in recent times [18–20]. An enrichment strategy is being adapted to design a synthetic microbial community in order to attain an optimal composition of microbial community with desired functional capabilities. The prevailing gut microbial community are subjected to adapt the environmental conditions which favors the species to accomplish the target functions.

In case of in vivo enrichment strategy a shift in the microbial community structure are caused by different feeding pattern of insects/ruminants (i.e., lignin rich or lignin poor biomass) and its association with LCB degradation capability is predominantly explored [21–24]. For example, an intimate co-evolution of termite gut microsymbionts (*Tsaitermes ampliceps*) and its host after feeding with different substrates (filter paper, corn stover and pine wood) were examined by high throughput 454 pyrosequencing. In this study, each feed has remarkably enriched certain taxa along with the prominent modification in the relative abundance and evenness of gut microbiota thereby, unveiling the symbionts assisted LCB digestion in termites [21]. Similarly*,* in order to discover the variants involved in the cellulose and hemicellulose breakdown system, Guerrero et al. [22] studied the influence of different LCB diets in *Anthonomus grandis* (Cotton boll weevils) gut. On characterizing the gut symbionts, the enrichment in the prokaryotic community structure and its hydrolytic activities showed a higher prokaryotic diversity and richness for the diets with more complex and variable composition.

An integrative omics analysis together with CAZymes characterization has been explored for understanding the holobiont (host and its resident microbes) level community adaptation of termite species (*Cortaritermes* sp.) after feeding with Miscanthus. Gut bacterial gene expression profile alternation and the transcriptional abundance of CAZymes advocates a shift towards increased utilization of cellulose and arabinoxylan by the host [25]. Likewise, upregulation of expression level of many CAZyme encoding genes, laccase (evgtrinloc15173t0 and evgtrinloc11252t0) genes and endoglucanase genes (evgtrinloc27093t1 and evgtrinloc16407t0) in the transcriptome of bamboo feed *Cyrtotrachelus buqueti* (bamboo snout beetle) were determined by high throughput RNA sequencing [19]. In vivo degradation efficiency of lignin, cellulose and hemicellulose component of bamboo were found to be 69.05%, 61.82% and 87.65% respectively through fecal component analysis. Therefore, in vivo enrichment unveiled the in vitro overexpression of lignocellulolytic enzymes for its efficient saccharification [26].

Similar to insects, diet type and feeding frequency of ruminants predominantly influence the microenvironment, where the unstable rumen microbiome undergoes a community dynamic turnover. Thus, exhibits a changes in the ionic strength, redox potential, fermentation time and rumen pH [27]. Effects of high forage to concentrate diet ratio towards rumen microbial community shift during the feeding cycle of dietary cow was investigated [23]. The study elucidates that the rumen microbiome is structurally similar, yet with distinct composition and thus aid in rumen metabolism manipulation. Whereas, based on next generation sequencing technique, no considerable effect on the rumen dynamic community composition was observed in Holstein heifers under 50:50 forage to concentration ratio with 8% dietary difference in energy level condition [28].

On the other hand, influence of different feeding methods (i.e., feedlot and grazing) on cellulolytic microbial abundance and composition of Tan sheep rumen has been recently explored [24]. A structural variation in the rumen bacterial population has been observed, where the cellulolytic bacterial abundance is decreased, yet increases the abundance of Succinivibrionaceae family associated with starch degradation. A sequential enrichment strategy was deployed in developing the efficient microbial consortia of ruminal and insect gut which displays the target bioconversion features. An in vitro LCB conversion system was established via sequential batch culture technique using ruminal microflora in order to mimic the rumen ecosystem [29].

A holistic analysis underlying the potency of in vitro enrichment of rumen microbes for its high capacity LCB degrading consortium was defined in terms of LCB degradation, dynamic behavior of syntropic microbes and concomitant production of fermentation end products (methane, Volatile Fatty Acids (VFA), hydrogen, acetate and CO_2_) [18, 29, 30]. A functionally stable rumen derived non-sterile microbial consortium with the ability of efficient LCB hydrolysis was attained only after a 10 sequential batch-reactor enrichment. A maximum amount of about 1.94 ± 0.04 g VFA/L was obtained using wheat straw as a sole carbon source over a period of 7 days [30]. Likewise, an enriched rumen derived co-culture of anaerobic fungi and methanogens resulted in a metabolically stable consortium for LCB bioconversion into fermentation end products after being subjected to a subsequent batch culture [18]. A rapid stabilization of rumen microbial community emphasizes the severity of enrichment culture conditions and thus endorses the major use of LCB as a sole carbon source in selecting a stable and robust microbial consortium for its bioconversion. Auer et al. [31], revealed the LCB degrading potency of xylophages termite gut microflora which includes an anaerobic bioreactor-based enrichment strategy for gut microbial community using wheat straw as a primary carbon source for its bioconversion into carboxylates.

Like other insects, *Helicoverpa armigera* Hubner (cotton bollworm), *Achatina fulica* (giant African land snail) and *Pachnoda marginate* (scarab beetle) harbors syntrophic gut symbionts which augments the host nutrition. Herein, subsequent enrichment for the prospection of gut microflora and enumeration of cellulolytic bacterial isolates based on LCB deconstruction was examined using various substrates (LCB, carboxymethyl cellulose (CMC) and xylan) respectively [32–34]. The comprehensive analysis of CAZy class perceived in rice straw enriched consortium of *Scirpophaga incertulas* (rice yellow stem borer). Metaexproteome examines a total of 55 Glycoside Hydrolase (GH) domains and thus performing the dynamics of CAZy protein expression by its hierarchical clustering at various timepoint [35]. On the other hand, Ali et al. [20] demonstrated the ordination clustering analysis of enriched microbial consortium from gut symbionts of *Coptotermes formosanus* (wood feeding termites). The study reveals a unique metabolic and physiological characteristic of the individual strains based on the lignocellulolytic enzyme activity, utilization of lignin monomers and thermotolerance ability. Therefore, in recurrent enrichment, the stability and dynamic structure of the gut microbial consortia revealed that the convergent adaptation of consortia would lead to a decrease in richness and diversity which are driven by the applied selective pressure during the enrichment process [36]. In order to characterize the species diversity in the gut and rumen microbial community, Shannon index was majorly used. Shannon index, also known as Shannon diversity index, mathematically measures the microbial species diversity within the gut/rumen community. Species richness (number of microbial species present) and evenness (relative abundance of different microbial species) are the two main factors majorly considered for characterizing the species diversity. Whereas, Chao 1 is a non-parametric richness estimator which simply measures the species richness based on the abundance data [37]. Based on the community structure analysis, the operational taxonomic units, Shannon index and Chao1 index were significantly higher in 0^th^ sub-cultivation (T0 sample) as compared to 10^th^ and 20^th^ sub-cultivation (T10 and T20 samples). Hence, the enrichment strategy by supplementing microbial consortium with rice straw and filter paper led to significant enrichment of cellulolytic microbes. Therefore, in vitro and in vivo manipulation of gut microbial community aided by high throughput techniques perhaps influences the structural compatibility of gut microbiota towards LCB degradation [38].

## Molecular characterization of microbial symbionts

Conventional culture dependent approaches are widely involved in enumerating the microbial and the functional diversity yet these approaches are unable to culture the uncultivable microbes of insects and rumen holobiome. Thus, the conventional techniques are being replaced by various DNA/RNA based molecular characterization techniques for the complex gut/rumen microbial communities. Therefore, the culture independent molecular approaches afford an extensive genomic/metagenomic perspective of microbial and functional diversity in various insects and ruminants.

### Culture independent molecular approaches

Development of next generation metagenomic and meta-omics approaches empowered a deep insight into the synergistic microsymbionts which intricates the microbial community characterization and its functional diversity. Proteomics and transcriptomics analysis are able to reveal the cellulolytic machinery of microbiome more efficiently, however the former uses LC–MS/MS in evolving the native protein database. It signifies the protein expression and its nature within the microbial community based on the database of closely related proteins [39, 40]. On the other hand, a comprehensive study of novel microbial protein via high throughput next generation sequencing are delivered from the abundant cDNA data from the long-read transcriptome sequencer. Whereas, gene expression levels of the microbial protein are being studied by short-read deep sequencer. In this context, Wang et al. [41] established an efficient cellulase characterization platform that combines secretome and transcriptome complimentary analysis. Herein, functional characterization of *Neocallimastix patriciarum* W5 (cow rumen fungus) was performed in order to accelerate the cellulase genes identification, classification and further its application in rice straw deconstruction.

Till date, several scientific literatures on insects transcriptomic profiles are available that are associated with the gut bacterial colonization. For instance, Scully et al. [42] defined the metagenome and meta-transcriptome of *Anoplophora glabripennis* (cerambycid beetle) gut bacterial community which highlights the holobiome influence on biomass digestion. Exploring the microbial communities of natural biomass utilization system (insects and ruminants) using high throughput metagenomic DNA sequencing provides a notable insight into the functional characterization of gut microbiome. First study on the functional characterization was performed by Suen et al. [43], where the metagenomic data of CAZymes from leaf-cutter ants microbiome revealed the contribution of diverse gut bacterial symbionts in the LCB degradation. The functional characterization of *Pseudacanthotermes militaris* (termite gut) metagenome discovered an array of multimodular xlyan degrading enzymes that belongs to GHF10. Based on the sequence analysis, an unusual organization of this enzyme domain was observed where the one enzymatic domain is intercalated with the two carbohydrate binding modules (CBM). Therefore, a in depth characterization of full length and truncated variants of *Pm*25 gene (Xyn10C-like enzyme- part of xylan utilization locus) was performed in order to understand the CBM role with unusual multimodularity. Results indicate that the CBM would act synergistically to improve the enzyme activity and its is more specific towards the xylan hydrolysis [44]. In turn, the metagenomic of gut symbionts sever as a strategy in novel enzyme identification that are potential with high enzyme activities. However, metagenomic approach assembles only a subclass of genes, where the average occurrence of target genes is inferior to the two GH gene for each bacterial genome.

Meta-transcriptomic, a functional annotation of RNA complements of whole microbial community governs the transcriptionally active microbial population and the genes transcribed within the holobiome. Thus, the functional profile of the microbial community aids in the identification of its role and the active metabolic pathway under certain environmental conditions [45]. Marynowska et al. [46] optimized the framework of hindgut prokaryotic meta-transcriptomics of termites in order to examine the lignocellulolytic potential of gut symbionts. The study revealed the overexpression of CAZyme (cellulose/hemicellulose degrading genes) within the symbiotic communities from the hindgut digestome via gut sampling approach and the prokaryotic based mRNA transcript enrichment pipelines. However, integration of meta-genomic and meta-transcriptomic data is required to examine the gene expression associated with the sequence conservation. Thus, it reveals the wide evolutionary outlines across the functional and taxonomic profile of diverse microbial communities [47].

### In situ metabolic activity of holobiome by stable isotope based techniques

**Fig. 2:**
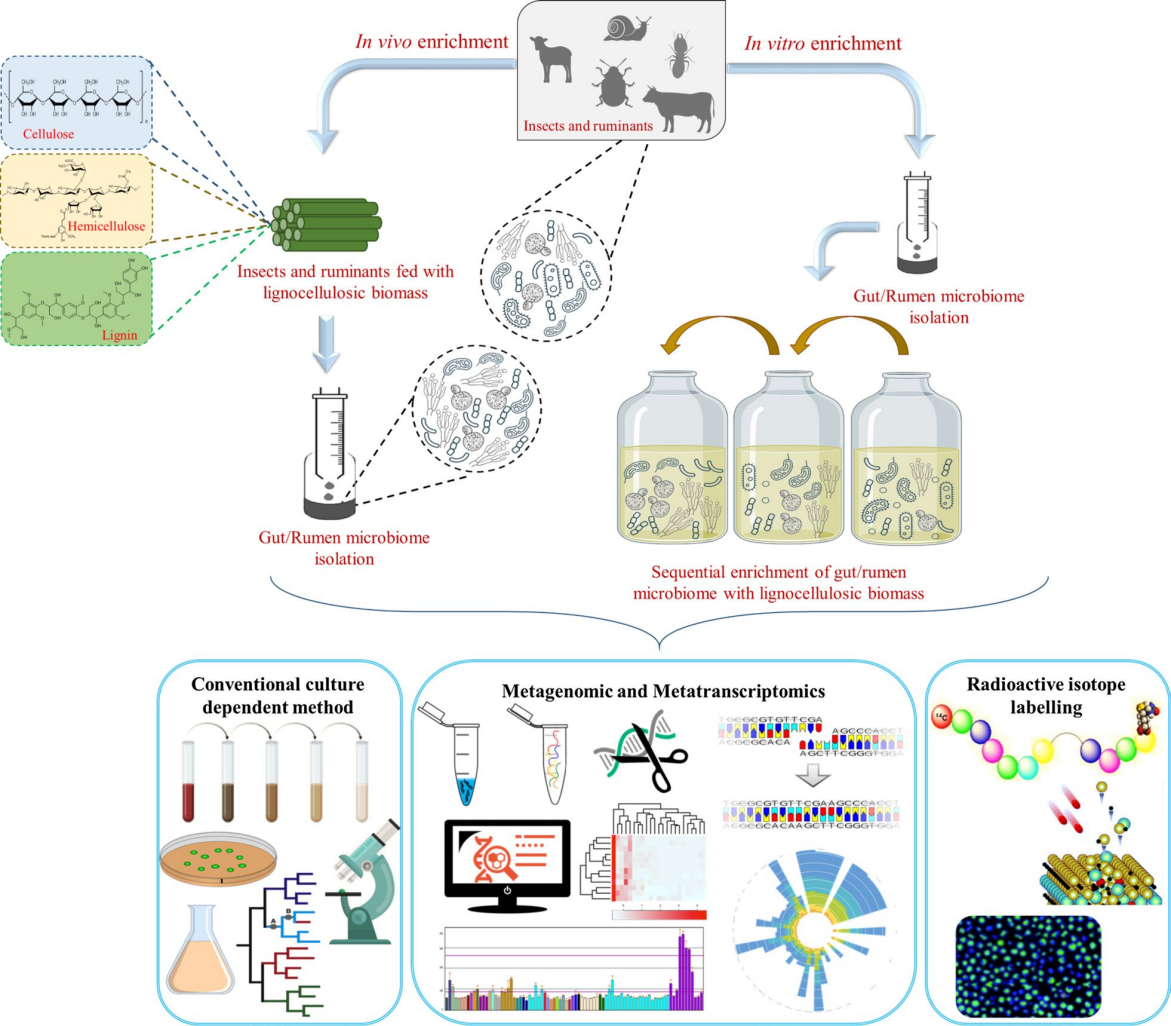
Schematic representation of gut/rumen microbiome enrichments and its molecular characterization techniques

Though many techniques are available for microbial characterization, yet the advent of single cell in situ stable/radioactive isotope based probing technique namely secondary ions mass spectrometry (SIMS), Raman micro-spectroscopy and micro-autoradiography could unleash the phylogenetic link between the metabolic function of unculturable microbiome [48]. These technologies would reveal the metabolically active microbes within the complex microsymbionts of various insects and ruminants, in turn adds an vital knowledge in modern microbial ecology [49–51]. Conceptually, Stable Isotope Probing (SIP) is a non-invasive and culture-independent approach, where the whole microbial community is being exposed to the highly enriched radioactive substrate i.e., ^13^C or ^15^ N in situ [52]. Deployment of an effective quantification strategy via combining SIP (^13^C-Cellulose) with NanoSIMS offers the competence of protist (harboring the hindgut of termite) to phagocytoses i.e., engulfing cellulose/wood along with the enzymatic degradation of ingested wood [53]. In turn, stable isotope enriched DNA/RNA was cloned into fosmid vectors for functional gene analysis in order to determine the microbial identity via 16S rDNA amplification. SIP seemed to be dependent upon the significant amount of heavily labelled radio isotope integration into nucleic acid isolated from rumens microsymbionts [50]. Unraveling the symbiotic bacterial networks of *Melolontha hippocastani* (forest cockchafer) involved in processing the recalcitrant diets and nitrogenous waste recycling via SIP-Illumina. It combines in situ SIP multiple trophic links (^13^C- cellulose, ^15^ N- urea) with Illumina MiSeq. Further, introducing the obtained metagenome to PICRUSt software would predicts the functional composition of gut bacteria which has the ability to degrade hemicellulose [54]. While, the ^13^C glucose SIP with pyrosequencing unveils the metabolic activity of *Spodoptera littoralis* (cotton leafworm) gut microbiota [55]. On the other hand, SIP could be used with molecular cytogenic Fluorescence in situ hybridization (FISH), which observes the functional and taxonomic microbial diversity along with the simultaneous identification of individual microbial cell and its substrate uptake quantity [56, 57]. Here the designed molecular FISH probes would target the 16S rDNA gene of diverse taxonomical communities. FISH is being performed either as a direct in situ hybridization on a gut bacterial isolate or on a *E. coli* clone carrying 16S rDNA of target uncultivable bacteria. For instance, localization of insect gut/ruminal symbionts through FISH was explored by Hayashi et al. [58]. A comprehensive analysis of PCR amplicon of 16S rDNA gene of insect gut symbionts via phylogenetic microarray hybridization showed a diverse range of probes that are designed based on the target gene of known bacteria. Hence, it recognizes the microbial taxonomic group within the symbiotic community of insects. Based on this, Scharf et al. [59] simultaneously studied the meta-transcriptome of gut symbionts isolated from *Reticulitermes flavipes* (termite). The author compared the meta-transcriptome composition of the termite gut across 5 categories (dietary, xenobiotic, immunological, hormonal and social) which delivers a new perception in co-opted eusocial gene in termite symbionts and its physiology. Hence, exploring the metabolic contribution of insects/ruminants holobiome encompassing the bacteria, protist, eukarya and archaea could elucidate more details about its single cells, community and the population behaviour. A schematic representation of in vivo and in vitro enrichment of rumen/gut symbionts and its culture-dependent and culture-independent molecular characterization techniques is depicted in Fig. 2.

## Microbial symbionts as a source for endogenous lignocellulolytic enzymes

A diverse range of lignocellulolytic enzymes are produced by the microbial symbionts for its notable adaptation to various dietary resources in order to overcome its host nutritional constraints. In case of inadequacy of its own metabolic repertoire or a novel niche colonized and the presence of refractory substrate in the host diet would lead to the production of beneficial gut symbiotic digestive enzymes [60]. The subsequent sections provide a deep insight into the production of lignocellulolytic enzymes from gut symbionts of various insects and ruminants (Table 1).

**Table 1:** Summary of lignocellulolytic enzyme production from various insects and ruminants gut microbes

S. no	Source	Substrate	Microbes	Enzyme activity			Conditions	Reference
				Laccase	MnP	LiP		
Ligninolytic enzymes								
1	Rubber termite (*Coptotermes curvignathus)*	Kraft lignin	*Lysinibacillus* sp., (novel)	70.67 U/L	76.36 U/L	196.07 U/L	SmF 30 °C, pH-7, 120 rpm and 5 to 7 days of incubation	[63]
			*Acinetobacter* sp.	50.74 U/L	49.39 U/L	262.49 U/L		
			*Bacillus* sp.	46.48 U/L	36.06 U/L	247.52 U/L		
2	Termite (*Anacanthotermes*)	Guaiacol	*Bacillus* sp. CF96	287.66 U/mL	–	–	SmF 37 °C, pH 8, 1% (v/v) inoculation and 4 days of incubation	[168]
3	Earthworm	Wheat bran	*Bacillus safensis* DSKK5	12.11 U/mL	–	–	SmF 37 °C, 200 rpm and 2 days of incubation	[62]
4	Termite (*Amitermes hastatus)*	Indulin AT (Kraft pine lignin)	*Streptomyces sp.* MV32	5.66 U/mL	–	–	SmF 30 °C, 160 rpm and 10 days of incubation	[65]
5	Termite (*Bulbitermes* sp.)	Saw dust	*Aspergillus* sp. A1	69.44 U/g	43.18 U/g	729.12 U/g	SSF 30 °C, pH 7.0 and 14 days of incubation	[67]
			*Bacillus* sp. B1	45.14 U/g	47.73 U/g	577.03 U/g		
			*Bacillus* sp. B2	71.18 U/g	41.48 U/g	500.99 U/g		
6	Cattle	Rapeseed	Rumen fluid microbial consortia	–	≈ 85 U/L	–	SmF 37 °C, 170 rpm and 1 day of incubation	[115]

S. No	Source	Substrate	Microbes	Enzyme activity				Conditions	Reference
				β-glucosidase	Endoglucanase	Exoglucanase	Xylanase		
Cellulolytic and Hemicellulolytic enzymes									
7	Termite (*Bulbitermes* sp.)	Saw dust	*Aspergillus* sp. A1	22.97 U/g	108.54 U/g	1.8 U/g	96.82 U/g	SSF 30 °C, pH 7.0 and 14 days of incubation	[67]
			*Bacillus* sp. B2	1.81 U/g	10.02 U/g	32.16 U/g	66.33 U/g		
			*Brevibacillus* sp. Br3	5.45 U/g	3.46 U/g	14.19 U/g	104.96 U/g		
8	Wood-feeding termite (*Reticulitermes chinensis)*	xylan or D-xylose	*C. pseudorhagii* SSA-1542 T	–	–	–	1.73 U/mL	30˚C and 3 days of incubation	[129]
9	African land snail (*Achatina fulica*)	Grass straw, CMC and Sugarcane bagasse	*B. tequilensis* G9	≈ 598 IU/mL	≈130.75 IU/mL	≈ 950 IU/mL	≈ 110 IU/mL	SmF 150 rpm, 37 °C and 8–14 days of incubation	[169]
10	Cotton bollworm (*Helicoverpa armigera)*	Saw dust	*Klebsiella* sp. MD21	78.45 IU/mL	258.93 IU/mL	13.41 IU/mL	276.71 IU/mL	SmF 160 rpm, 37 °C for 14 days	[34]
11	Termite *(Coptotermes formosanus)*	Sawdust	*Dyella sp.* SSA-1562 T (Cf-S-11)	6.52 U/mL			–	SSF 35 °C, pH 7 and 5 days of incubation	[20]
			*Vanrija humicola* SSA-1544 (Cf-S-17)	6.06 U/mL			–		
			SSA-6 microbial consortium	15.96 U/mL			14.6 U/mL		
12	Earthworm (*Perionyx excavatus*) *Glyphidrilus spelaeotes*	cellulose	Bacterial isolates	0.42 to 0.59 µM glucose/mL /min			–	SmF 30 °C and 2–28 days of incubation	[170]

*SSF* Solid State Fermentation, *SmF* Submerged Fermentation; Manganese Peroxidase, *LiP* Lignin Peroxidase

### Ligninases

Apart from fungi (white rot/brown rot)—a conventional ligninase producers, a vast variety of wood feeding insects have evolved the complex ecosystem and its ability to degrade the recalcitrant lignin for holocellulose accessibility still remains unexplored.

For the bygone year, gut bacterial microsymbionts from the wood feeding termites and invertebrate animals like earthworm has gained a ubiquitous interest for the production of endogenous ligninases that are primarily classified as peroxidases (manganese peroxidase and lignin peroxidase) and laccase [61, 62]. The endogenous ligninases are identified in the guts and the salivary glands of the insects which are involved in the oxidation and polymerization reactions. Gut bacterial isolates namely *Bacillus safensis* DSKK5, *Lysinibacillus* sp., (novel), *Acinetobacter* sp., and *Bacillus* sp., from earthworm and *Coptotermes curvignathus* are the potential ligninase producer under submerged fermentation. Among them, the novel lignin degrader (*Lysinibacillus* sp.,) showed a maximum activity of about 70.67 ± 16.82 U/L and 76.36 ± 15.74 U/L of laccase and manganese peroxidase, respectively [62, 63].

Termites harbor the gut symbionts with the synergistic mechanism for their wood digestion yet, the mechanism of lignin degradation by the endogenous ligninase from the gut microbiota was not clearly defined. The meta-transcriptomic analysis of *Reticulitermes flavipes* (lower termites) revealed the presence of various endogenous host ligninases like phenol oxidases, laccases, cytochrome P450s and esterases. The functional analysis of laccase gene expression and phenol oxidase activity are more likely to be restricted in the salivary gland and foregut (symbiotic free) respectively. Further, the phenol oxidase activity induced by lignin would serves as a potential pretreatment enzyme for the industrial LCB bioprocessing [64]. On the other hand, the exploitation of actinobacteria with the diverse metabolic capability was hardly exploited for its isolation from the termites gut microenvironment. Some of its isolates namely *Odontotermes*, *Micromonospora* sp., *Microcerotermes, Streptomyces* sp*., Macrotermes* and *Amitermes* are prevalent among the both higher and lower termites. Nevertheless, its biological role and the production of certain oxidases is largely unspecified in lignin degradation. Le Roes-Hill et al. [65] discovered actinobacteria as a novel oxidative enzyme producer that are isolated form the hindgut of the *Amitermes hastatus* (higher termites) with unique ability to metabolize indulin (Kraft pine lignin) as a sole carbon source. The aforementioned potent strain for oxidative enzyme production was subjected to the 16S rRNA gene analysis for its identification. Recently, Xiao et al. [66] has investigated the multiple enzymatic properties of laccase derived from hindgut microbes of fungus growing termites (*Macrotermes barneyi.*). In this study, *BaCotA*-laccase gene was isolated and cloned into E. *coli* and thus the recombinant gene was thermostable, alkali and organic solvent resistant with a specific activity of about 554.1 U/mg at an optimum temperature and pH (70 ºC and 5.0).

The capability of bacterial-fungal isolates as mixed culture from the microflora of *Bulbitermes* sp*.* has been reported to produce cocktail ligninolytic enzymes upon metabolizing the untreated saw dust substrate through solid state fermentation (SSF). Mixed culture of isolates such as *Bacillus* sp*.,* B2 and *Brevibacillus* sp.*,* showed a better volumetric productivity of lignin peroxidase (362.51 U/L.h) that was significantly higher in SSF [67]. Similarly, exploration of enriched termite gut microbial consortium for the production of cocktail ligninase showed a higher ligninolytic activity of about 308.3% when compared with the single strain isolates [20]. Thus, the improved production of ligninase cocktail enzyme from insect gut microbiota are of great interest for its responsibility in LCB depolymerization or lignin removal.

### Cellulase/hemicellulases

Cellulase from the gut symbionts has been identified in 68 insects species associated with 8 diverse taxonomic orders that are responsible for the cellulose assimilation. For decades, insects are being considered as a great cellulase producer owing to its more tolerance and stability in cellulose bioconversion. Insect cellulases are being encompassed with 48 GH families, thus advocating its vital role in insect cellulose assimilation system [43, 64, 68].

Many herbivorous insects like termites, silkworms, snails and beetles have been explored to identify the syntrophic symbionts in their gut microflora which are responsible for the hydrolysis by its complex cellulase known as cellulosome. In animals, genes encoding the endogenous enzymes being involved in plant cell wall degradation are attained by horizontal gene transfer mechanism [69]. Endogenous cellulase from wood feeding insects mainly depend upon the microsymbionts that are being involved in the breakdown of LCB they feed upon [69, 70]. A high homology has been revealed by the distantly related insects for endo-β-1,4-glucanases, indicates a universal cellulase gene distribution among the endoglucanase secreting endogenous insect species and this gene expression level differs over insect life cycle as ascertained in *Reticulitermes* termite species [71, 72].

Termites are the potent source of cellulose digester that are classified as symbiotic protozoa (lower termites—*Coptotermes gestroi, C. formosanus, Reticulitermes speratus,* and *R. flavipes,*) and non-symbiotic protozoa (higher termites—*Nasutitermes* sp.). The synergistic action of termites advocates its secreted β- glucosidases and endoglucanases which belongs to Glucose Hydrolase Family (GHF) 1 and GHF9 respectively. The gene expression for endogenous cellulase was observed in the midgut of the higher termites and the salivary glands of the lower termites. To add on, bacterial cellulase of termite gut isolates are being considered as a latent candidate for its robustness, versality, stability, showing multi-enzyme complexes, high growth rate, recombinant capacity and also for its thriving ability under extreme conditions. For instance, *Bacillus* sp., isolates of *Anacanthotermes* and *Bulbitermes* sp., termite is found with high cellulolytic activity, where the former secretes a novel β-1,4 glucanase with high specific activity of about 10.80 U/mg upon 8.85-fold purification [67, 73]. While *Bacillus* sp., isolates of *Bulbitermes* sp., exhibited endoglucanase activity—138.77 U/g, exoglucanase—32.16 U/g and xylanase—104.96 U/g [67].

Unlike termites, various natural host bioreactor such as *Antheraea assamensis* (silkworm), *Achatina fulica* (African land snail) and *Helicoverpa armigera* (cotton bollworm) would possess the host gut symbionts which colonize to effectively metabolize the recalcitrant diets. Yet the exploration of its critical interplay existing between the host-gut microflora-plants are meagre. Putative cellulolytic bacterium strains namely moni strain MGB05- (*Antheraea assamensis*) [74], *Bacillus tequilensis* strain G9- (*Achatina fulica*) [75] and *Klebsiella* sp. MD21 (*Helicoverpa armigera*) [34] with maximum cellulolytic enzyme activity from the aforementioned insects has been identified. A mixed culture of fungal-bacterial isolates has enhanced the endoglucanase, xylanase and β-glucosidase activity and its volumetric productivity when compared with the monocultures. This behavior would suggests that the fungal-bacterial consortium as a potent candidate for the production of cellulolytic cocktail enzymes [67].

### Lytic polysaccharide monooxygenases

Recently, characterization of lytic polysaccharide monooxygenase from insects gut symbionts has gained interest owing to its pivotal role in polysaccharide breakdown particularly cellulose, hemicellulose and chitin. This copper dependent endogenous enzyme catalysis the oxidative cleavage of glycosidic bonds in the recalcitrant polysaccharides to form monosaccharides. Cairo et al. [76], investigated the identification and functional characterization of two LPMOs in Coptotermes gestroi (lower termites) which belongs to the AA15 family (Auxiliary Activity) from CAZyme database. In addition to termites, Thermobia domestica gut were identified with more than 20 members of endogenous uncharacterized LPMO family that could oxidise cellulose. This study revealed that out of 23 full length peptide sequence encoding the LPMO catalytic domain, 21 were found in major amount based on the gut transcriptome analysis [77].

In a nutshell, the capability of single insect system secreting diverse lignocellulolytic enzyme favours to establish an enzyme complex that could be used in sugar alcohol industries.

## Mining LCB degrading genes of insects and ruminants gut symbionts

Gut microbial diversity is directly associated with the diet specialization and distinct stages in the life cycle of each species. This would reflect in lignocellulolytic enzyme diversity and further ends in stimulating the enzyme bioprospection in the utmost diverse circumstances.

Multi-enzyme complex being encoded by the fibrolytic gene clusters are extensively explored in two different paradigms, namely cellulosome and polysaccharide utilization loci-like system. Several scientific studies have reported the metagenomic based insights on rumen lignocellulolytic enzymes diversity from microsymbionts of various ruminants (Jersey cow, Angus cattle, yak and reindeer) [78–81]. Using comprehensive metagenomic and homology-based annotation, Jose et al. [82] deciphered the diversity of CAZyme in Holstein–Friesian crossbred Indian cattle that fed upon finger millet straw. In this study, authors have identified the candidate gene encoding the fibrolytic enzymes which includes diverse class of carbohydrate binding modules (contigs-1975), carbohydrate esterases (contigs-4945), glycoside hydrolases (contigs-11,010), polysaccharide lyases (480 contigs), auxiliary activities (contigs-115) and glycosyltransferases (contigs-6366). Among the CAZyme diverse group, glycoside hydrolases family was predominantly abundant (high total number of contigs) in rumen cattle metagenome due to its more complex LCB degradation system. To add on, genus *Prevotella* is the most abundant rumen bacteria which plays a significant role in diet shift from high calorie to high fiber. And this genus also contributes a significant proportion of more than 36% of CAZymes [83, 84] and well-known degrades of starch, hemicelluloses, and sugars [85, 86].

Tremendous efforts have been made in understanding the termite digestion system, yet the knowledge about its enzymology and its fibrolytic genes underlying the effective polysaccharide utilization mechanism of gut symbionts is inadequate. On account of this, Liu et al. [87] provided a deep insight in highly abundant multiple fibrolytic gene clusters and the cellobiose metabolism pathway of *Globitermes brachycerastes.* In this study, 50,000 fosmid clones was functional screened followed by pyrosequencing of 173 functionally positive clones. And also, putative CAZymes encoding saccharolytic gene operons (219) were identified via in silico analysis. Further, understanding the biochemical properties of those gene clusters, a 3 gene cluster of cellobiose metabolism and 1 xylanase gene were cloned and heterologously expressed in *E. coli* BL21. Herein, recombinant xylanase GH10 exhibited > 80% of maximum activity at an optimal temperature of about 38–65 ºC and retains > 60% of maximum activity under optimal pH- 5.5–9.5. Similarly, in order to validate the gut metagenomic assembly of *Arion ater* (common black slug), a predicted β-glucosidase gene was amplified and then expressed into *E. coli* where the recombinant protein (C-terminal His-tagged) was evaluated by western blot [88].

Upon meta-exoproteome and heterologous recombinant expression in *E. coli,* Singh et al. [35] identified the unknown catalytic counterpart of several novel CAZy protein from the gut microbial symbionts of rice yellow stem borer enriched with rice straw. In this study, one of most abundant xylanase annotated gene belonging to GH10 family was identified based on maximum emPAI score. Later it was cloned into pET30a expression vector and the purified recombinant xylanase showed a maximum activity at optimum temperature of 60 ºC, pH-7.0 with 72.2 µmol of reducing sugar/min/mg of V_max_ and 2.859 mg/mL of K_m_ against beechwood xylan.

Meanwhile, as the uncultivable gut protist being a large resident of wood feeding termites, the hemicellulase and cellulase gene in GHF (5,7 & 11) from *Coptotermes formosanus* are also cloned. Yet, discovery of an independent gene signifying the lignocellulolytic mechanism in protist community is inadequate [89–92]. Based on this, two independent studies were conducted on *Reticulitermes flavipes* and *Reticulitermes speratus* where the abundance of GH expression was observed via cDNA clone sequencing in protistan community [64, 93]. Xie et al. [94] studied the meta-transcriptomic profiles of functional gene being expressed in protistan community of *Coptotermes formosanus.* Herein, 454 pyrosequencing of enriched cDNA resulted in 223,477 reads, followed by de novo sequence assembly and Kyoto Encyclopedia of Genes and Genomes (KEGG) annotation which identifies 118 GH gene belonging to 18 diverse GHFs. Further, heterologous expression of xylanase (GHF10) in *Pichia pastoris* and the characterization of purified recombination protein (*xyl726-* 34 kDa) showed a specific activity of about 80.3 ± 3.1 U/mg of against birchwood xylan with 9.2 mg/mL K_m_ and 107.4 U/mg V_max_. Apart from gut protist, metagenomic DNA of free-living gut microbiota such as *Clostridiales, Bacteroidales*, *Enterobacteriales*, *Lactobacillales, Xanthomonadales, Pseudomonades, Spirochaetales, Actinomycetales, Desulfovibrionales, Bacillales, Burkholderiales, Synergistales* and 1460 species in *Coptotermes gestroi* (lower termite) has been subjected to Illumina-based de novo sequencing and followed by mining of LCB degrading gene in gut bacteria [95]. In concern with industrial application, a three-fold increase in the extracellular endoglucanase activity was able to attain in recombinant heterologous host (0.51 ± 0.02 μmol/min/mL) as compared to wild type (0.12 ± 0.01 μmol/min/mL) of *Bacillus subtilis* UMC7 isolated from *Macrotermes malaccensis* gut. The recombinant endoglucanase (56 kDa) which are highly specific to amorphous cellulose was active at optimum pH (6.0) and temperature (60 ºC) with an optimal enzyme activity (0.50 ± 0.01 μmol/min/mL) and further its activity was enhanced by Ca^2+^ ions addition [49]. Yet, another novel endo-β-1,4-glucanases highlighting the cellulase bifunctional was characterized from *Coptotermes formosanus* transcriptome [96]. The identified novel endogenous cellulase gene-CfEG5 belonging to GHF9 family was similar to CfEG3a gene yet, unlikely as an allelic variant as described in the former study [97]. The CfEG5 gene identified from the *Coptotermes formosanus* EST library, was expressed in *E. coli* using pET28a vector plasmids and thus the recombinant CfEG5 (rCfEG5) protein generated exhibits cellulolytic activity against filer paper, CMC and cellodextrins. Herein, the filter paper cellulose hydrolysis end product was primarily cellodextrins namely cellotriose and cellobiose. Upon mining, rCfEG5 gene showed a highest cellulolytic activity at an optimal pH 5.6 and temperature ranging between 37 ºC – 42 ºC. Whereas, the enzyme specific activity was in similar with rCfEG3a, however, rCfE5 lacks its activity against xylose and glucose with β-1,3/1,6 linkages. On comparison with other termite species, K_m_ values for rCfEG5 (5.6 mg/mL) and rCfEG3a (2.2 mg/mL) endogenous cellulase gene were found to be higher than the NtEG (4.67 mg/mL) and RsEG (2 mg/mL) in *Nasutitermes takasagoensis* and *Reticulitermes speratus* respectively.

**Fig. 3:**
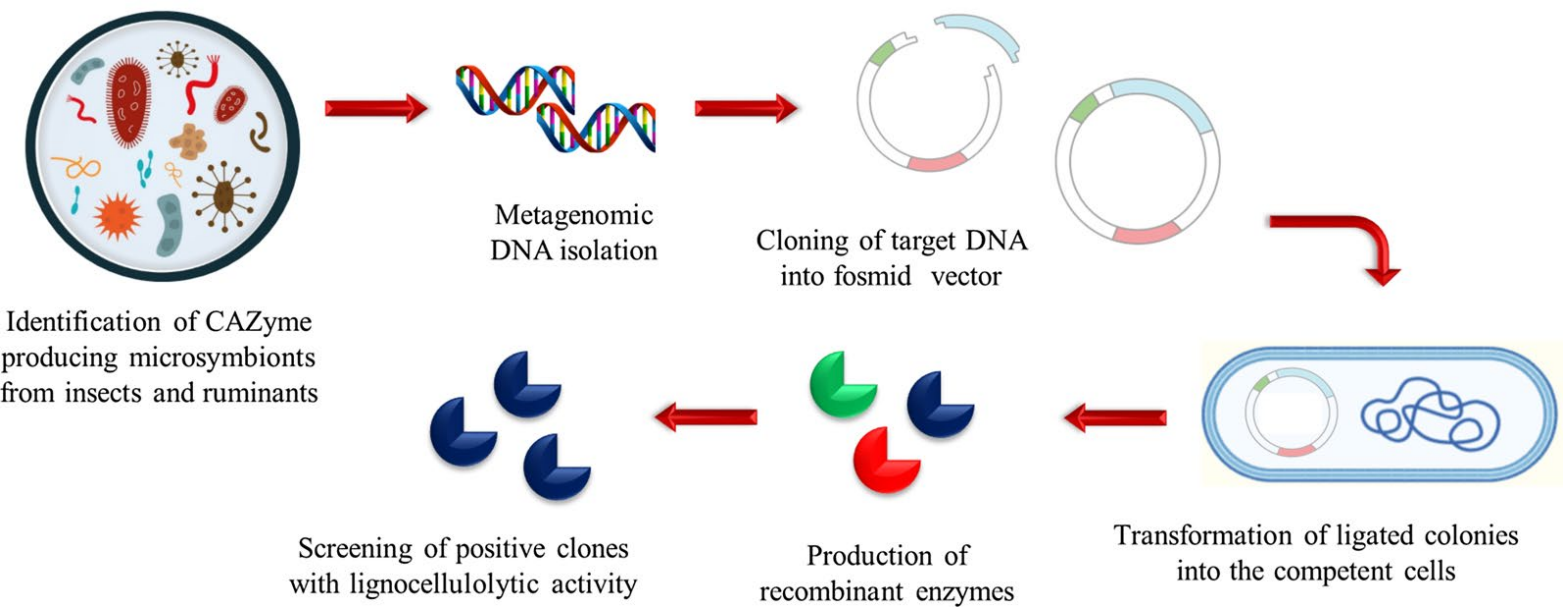
Steps involved in mining of LCB degrading genes of insects and ruminants gut symbionts

Recombinant CfEG5, produced in *E. coli*, was active against filter-paper cellulose and results in cellobiose and cellotriose which was similar to the enzymatic and biochemical properties of CfEG3a. These findings would lead to further investigation of both the evolutionary origin and relationships existing between the eukaryotic cellulase genes and the termite species. Figure 3 shows the mining of LCB degrading genes through metagenomic and meta-transcriptomic analysis of insects and ruminants gut symbionts which provides a deep insight into its plant biomass digestion system and further identification of lignocellulolytic biocatalyst beneficial for biofuel production.

**Fig. 4:**
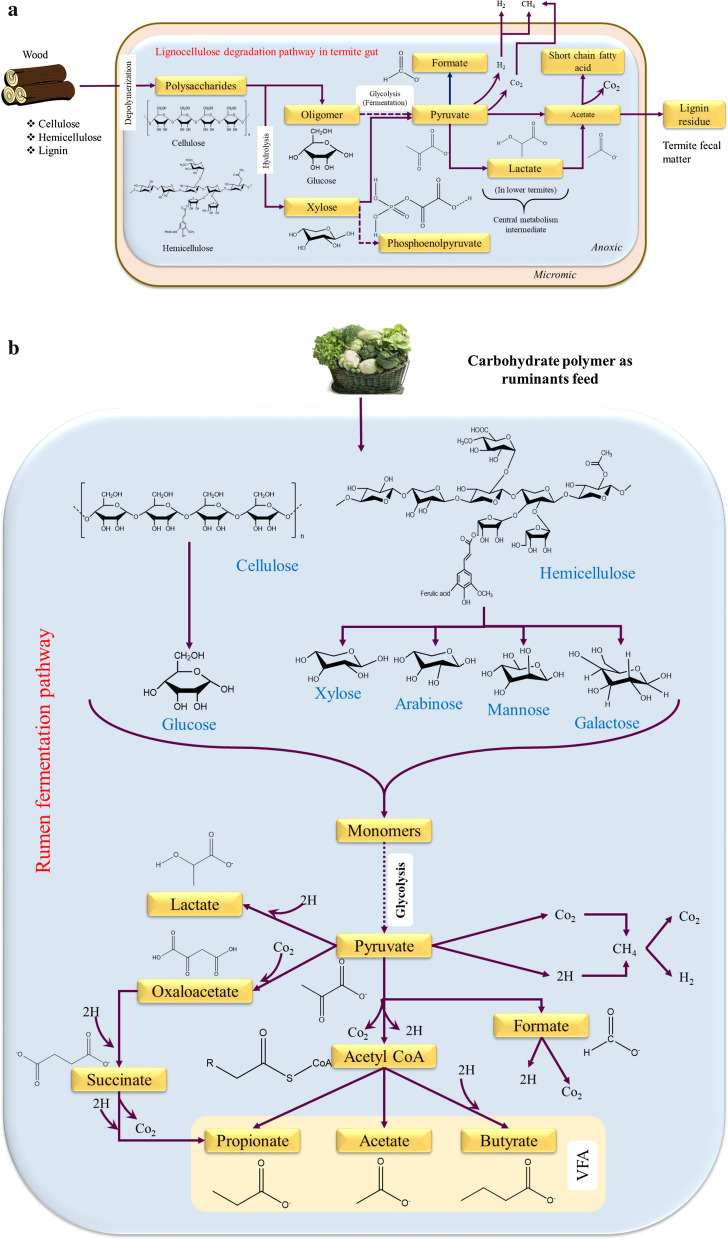
a Metabolic pathways within the insects gut holobiome community. **b** Metabolic pathways within the ruminants gut holobiome community

## Role of microbes and different process strategies adopted for biofuel production

In nature several gut/rumen microbial ecosystems are well known for the co-existence of symbiotic microflora, where the annotation of microbial dynamic interaction and its enzyme profile remains vital in commercial biofuel application. Figure 4a and b represents the microbial metabolic pathway of insects and ruminants that aids in better understanding of its gut microbial role in biofuel production. The subsequent section has been dealt to provide an insight into different process strategy that are adapted in the LCB bioconversion which includes depolymerization of lignin/lignin model compounds, ethanol, hydrogen, biogas and carboxylates production (Table 2).

**Table 2:** Summary of biofuels and valued added chemicals production from insects and ruminants gut microbes

S. no	Source	Microbes		Substrate	Methane	VFA	Hydrogen	Ethanol	Others	Reference
Insects										
1	Termites (*Termes hospes, Nasutitermes ephratae, Microcerotermes parvus, and Nasutitermes lujae*)		Microbial consortium	Wheat straw	–	2.2 to 5.8 g/L	–	–	–	[31]
2	Termite (*Nasutitermes ephratae*)		Microbial consortium	Wheat straw	–	179.3 mCmol VFA/L	–	–	–	[167]
3	Termite (*Odontotermes formosanus)*		Co-cultures of *Bacillus* and *Clostridium* sp.	Glucose	–	–	4.08 mmol/mL	–	–	[136]
				Unautoclaved mango tree substrate	–	–	1.605 mmol/mL	–	–	
				Cellulose	–	–	1.42 mmol/mL	–	–	
				Humic acid	–	–	0.706 mmol/mL	–	–	
4	Termite (*Globitermes* sp.)		*Enterobacter cloacae* KBH3	Glucose	–	–	7.4 mmol/L/h	–	–	[139]
5	Termite (*Nasutitermes lujae*)		*Clostridium termitidis* strain CT1112	Cellobiose	–	–	4.6 mmol/L or 0.26 mmol/L/h	3.7 mmol/L or 0.18 mmol/L/h	Acetate—5.9 mmol/L Lactate—2.0 mmol/L Formate—4.2 mmol/L CO_2_—5.6 mmol/L	[140]
			*Clostridium termitidis* strain CT1112	α-Cellulose	–	–	7.7 mmol/L or 0.14 mmol/L/h	3.1 mmol/L or 0.06 mmol/L/h	Acetate—7.2 mmol/L Lactate—0.5 mmol/L Formate—2.9 mmol/L CO_2_—6.9 mmol/L	
6	Termite (*Cubitermes speciosus*)		*Clostridium mayombei* sp.	Glucose	–	–	13.4 mmol/100 mmol of substrate	–	Acetate—251.5 mmol/100 mmol of substrate CO_2_—6.7 mmol/100 mmol of substrate	[171]
				Xylose	–	–	–	–	Acetate—206.4 mmol/100 mmol of substrate	
7	Termite (*Pterotermes occidentis*)		*Acetonema longum gen*	Glucose	–	–	88.9 mmol/100 mmol of substrate	–	Acetate—17.3 mmol/100 mmol of substrate Propionate—10.2 mmol/100 mmol of substrate CO_2_—137.0 mmol/100 mmol of substrate	[142]
8	Superworm (*Zophobas morio*)		Midgut extracts	microcrystalline cellulosic substrates MCC 20	–	–	–	–	Reducing sugar—2.480 mg/mL	[122]
			Larvae	MCC 20	–	–	–	–	Reducing sugar—0.399 mg/mL	
9	*Tipula abdominalis* larvae		Cellulolytic bacterial isolate 27C64 + *S. cerevisiae*	Pre-treated pine	–	–	–	29.8 g/L	–	[127]
10	Termite (*Coptotermes formosanus*)		*Meyerozyma guilliermondii* SSA-1543 T*, Vanrija humicola* SSA-1544, *Starmera dryadoides* SSA—1549 T, *Acidisoma tundra* SSA-1560, *Dyella* sp. SSA-1562 T and *Burkholderia* sp. SSA-1567 T *Saccharomyces*	Saw dust	–	–	–	25.5 g/L	–	[20]
			*Meyerozyma guilliermondii* SSA-1543 T		–	–	–	13.9 g/L	–	
			*Vanrija humicola* SSA-1544		–	–	–	8.4 g/L	–	
11	Termite *(Cryptotermes brevis)*		*Bacillus* sp. BMP01	Wheat straw	–	–	–	–	Reducing sugar—439 mg/g Saccharification efficiency—51.95%	[61]
			*Ochrobactrum oryzae* BMP03 strain		–	–	–	–	Reducing sugar—360 mg/g Saccharification efficiency- 42.60%	
12	Termite gut (*Reticulitermes chinensis*)		*Candidapseudorhagii* sp. nov. strain SSA-1542 T	D-xylose	–	–	–	14.7 g/L or 0.31 g/g	–	[129]
			*Hamamotoa lignophila* sp. nov. strain SSA-1576 T		–	–	–	10.1 g/L or 0.22 g/g	–	
13	Beetle		*Spathaspora passalidarum*	Xylose	–	–	–	0.41 g/g	–	[172]
				Xylose + Cellobiose	–	–	–	0.43 g/g	–	
14	Beetle		*S. stipites NRRL Y-7124*	Xylose	–	–	–	0.39 g/g	–	[173]
			*S. passalidarum* *NN245*		–	–	–	0.43 g/g	–	
15	Scarab beetle		*Promicromonospora pachnodae* sp.	Glucose or xylose	–	–	–	2.3–2.8 Mm	Lactate-2.9–3.0 mM Formate-0.5–0.7 mM	[32]
Ruminants										
16	Sheep Rumen		Cellulolytic bacterial culture	Wheat Straw	196 mL_N_ CH4/g VS	–	–	–	–	[151]
17	Bull rumen		Rumen fluid microbial consortium	Wheat Straw	288.2 mL/g VS	–	44.6 mL/g VS	–	Propionate—22.19 mmol Acetate—183.4 mmol	[152]
	Heifer rumen				213 mL/g VS	–	–	–	–	
18	Deer rumen		Rumen fluid microbial consortium	Glucose	-	–	52 mol/100 mol of substrate	17 mol/100 mol of substrate	Acetate—30 mol/100 mol of substrate Lactate—12 mol/100 mol of substrate	[134]
				Fructose	–	–	43 mol/100 mol of substrate	20 mol/100 mol of substrate	Acetate—70 mol/100 mol of substrate Lactate—13 mol/100 mol of substrate	
				Ribose	–	–	54 mol/100 mol of substrate	3 mol/100 mol of substrate	Acetate—77 mol/100 mol of substrate Lactate—6 mol/100 mol of substrate	
19	Cattle rumen		*Clostridium xylanolyticum, Clostridium papyrosolvens, Clos*—*tridium beijerinckii, Ruminococcus sp., Ethanoligenens harbinense, and Desulfovibrio desulfuricans*	Napier grass	–	–	Around 60 mL	–	Reducing sugar—around 0.20 mg/mL	[29]
20	Cattle rumen		*C. puniceum* Ru6	Glucose	–	2634 mg/L	3379.2 mmol	Ethanol—126.6 mg/L	Butanol—42.4 mg/L	[131]
			*C. xylanolyticum* Ru15	Glucose	–	1728 mg/L	1970.6 mmol	3418.1 mg/L	Butanol—47.4 mg/L	
			*C. xylanolyticum* Ru15	Napier grass	–	1405 mg/L	–	1626 mg/L	–	
			*C. puniceum* Ru6	Napier grass	–	1667 mg/L	–	72. 8 mg/L	–	
21	Cattle		Rumen fluid	Rapeseed	1901.7 mL of CH_4_ reactor^−1^	Propionate—2.88 g/L Acetate—8.10 g/L	–	–	–	[115]
22	Cattle		Rumen fluid	Corn stover	–	13,271 mg/L	–	–	–	[116]

### Depolymerization of lignin/lignin model compounds

Lignin depolymerization is a critical oxidative phase preceding the carbohydrate digestion, which involves the release of entrapped polysaccharides and thus enables the accessibility of cellulosome hydrolyzation in LCB conversion to biofuels. Unlike other conventional depolymerization strategy such as physical and mechanical being the most affluent and high energy consuming process. Biological depolymerization naturally encompasses the lignin oxidation by extracellular hydrogen peroxide that are generated by auxiliary enzymes i.e., ligninases. Hence, it requires less energy to enhance the suitability of LCB for subsequent saccharolysis [98, 99]. However, biological depolymerization are being considered as a slow process owing to its low hydrolytic rate. Yet, the exploration of some characteristic wood feeding termites/snout beetles and ruminants gut symbionts possessed with capability of overwhelming the recalcitrant lignin barrier are discussed in the subsequent sections.

#### Insects microbiota allied depolymerization

To date, several insects species such as wood feeding termites, beetles, leaf cutting ants and wood wasps have been explored for the lignocellulose biodegradation ability [100]. Many biodegradation studies comprise the use of various lignin model compounds rather than any LCB source owing to its complex structure. For instance, β-O-4 bond linkage in the guaiacylglycerol-β-guaiacyl ether (lignin model compound) was almost 60% similar to inter-monometric bond linkage in softwood lignin. Depolymerization of lignin model compounds by novel bacterial gut isolates of *Nasutitermes takasagoensis* (*Burkholderia cepacia* KK01) and *Odontotermes obesus* (*Trabulsiella* sp.,) was evaluated. In this study lignin dimers such as dehydrodiconiferyl alcohol (phenylcoumarane bond), dehydrodivanillic acid (biphenyl bond), erythron-1,2-Bis (4 hydroxy-3-methoxyphenyl)-1,3 propanediol (β-1 bond) [101] and guaiacylglycerol-β-guaiacyl ether (β-O-4 bond) are used as a sole carbon source [101, 102]. *Odontotermes obesus* gut isolates showed a maximum degradation efficiency of about 60% for the guaiacylglycerol-β-guaiacyl ether upon 28 days of treatment, yet the *Burkholderia cepacia* KK01 gut bacterial isolates resulted in 61% to 94% of lignin degradation for all the dimer model compounds. Further this study revealed its degradation ability of monomer lignin models like phenol guaiacol and vanillic acid. Tsegaye et al. [61] studied the wheat straw depolymerization with the bacterial isolate *Ochrobactrum oryzae* BMP03 of wood feeding termites as a consortium with *Bacillus* sp. BMP01 (polysaccharide hydrolyzing strain) which resulted in 44.47% of lignin depolymerization.

In vivo degradation of various aromatic compounds (lignin model monomers and dimers, dyes and lignin sulfonate) and its modification phenomenon of *Coptotermes formosanus* revealed the metabolizing capabilities of wood feeding termites and the degradation rate was higher in foregut and midgut rather than hindgut [103]. Azure dye employed in lignin peroxidase assay are converted to veratraldehyde and phenol red involves the free radical generation upon ligninolytic enzymes action. Monomeric lignin model compounds like veratryl alcohol and vanillic acid were predominantly oxidized by lignin or manganese peroxidase that are similar to lignin sulfonate polymer from kraft pulping [101]. Whereas, in case of lignin dimers (desoxyanisoin (4,40-dimethoxydeoxybenzoin), benzylvanillin (4-benzyloxy-3-methoxybenzaldehyde) and 2,20-biphenyldiol), the biodegradation was implied when catalysed by bacterial isolates of xylophagous termite gut [104]. Majority of the aromatic compound degradation by the bacterial isolates and its modifications like decarboxylation and double bond reduction in side chains were observed under aerobic and anaerobic conditions respectively. The required oxygen is being supplied by aerated gut paunch epithelium for the aromatic ring cleavage [105]. Though, in vitro degradation for several aromatic compounds by termite gut microflora has been reported by many researchers, yet the utilization of cellulose despite the complex depolymerization mechanism are still remained to be unsolved.

Several studies on termite gut system on LCB digestion has suggested a novel physiochemical process are being involved in *Coptotermes formosanus*. Li et al. [106] have examined the physicochemical changes in the Chinese red pine biomass after passing them into the *Coptotermes formosanus* gut digestive system. In this study, the termite colony fed with sapwood blocks are dissected into separate gut segments to collect the sapwood particles in various phases of assimilation. Scanning electron microscopy (SEM) images revealed the lignin decarboxylation, side-chain oxidation of the termites midgut that leads to the lignin degradation. And thus, the free symbionts in midgut plays a critical role either in loosening or removing the lignin altogether. Less amount of lignin depolymerization was observed at 26 ºC possibly due to the oxidation in propyl side chain, ring methoxyl group demethylation, demethoxylation (spatial rearrangement) and also likely due to mechanical process like mandible mastication and gizzard grinding. Taken together, ligninolytic enzymes of termite gut symbionts in *Coptotermes formosanus* might be the vital factor in plant cell wall breakdown.

Although, most of the research on wood feeding insects suggests that many insects feed on predegraded wood which have exosymbiotic relation with fungi to overcome the lignin barrier. Yet, there are certain insect species that feeds on the healthy living inner woods. In this regard, Geib et al. [107] determined the fate of live wood feeding insects using ^13^C tetramethylammonium hydroxide thermochemolysis. In this study, a significant level of side chain oxidation and methoxyl group ring demethylation were detected in *Zootermopsis angusticollis* (lower termite) and *Anoplophora glabripennis* (beetle). Predominant ring hydroxylation with lignin degradation products such as 3,4,5-trimethoxybenzaldehyde (syringyl) and 3,4-dimethoxybenzldehyde (guaiacyl lignin) were observed distinctly in the termites species. Some researches were also performed to determine the role of *Cyrtotrachelus buqueti* (snout beetle) in LCB biodegradation. For instance, Luo et al. [26] investigated the gut bacteria isolates from snout beetle larva and adult (male and female) for LCB degradation. In vitro studies were carried out by utilizing the gut microflora for treating bamboo shoot particles over a period of 6 days. The hierarchy of lignin degradation efficiency was found to be larva (32.97%) > male adult (24.30%) > female adult (19.83%) that are consistent with the morphological changes observed after pretreatment. Recently, an anaerobic bioreactor based lignin deconstruction of wheat straw biomass was investigated by Dumond et al. [108] using the gut microbial isolates of various higher termites. Results revealed that up to 37% of lignin has been removed after digestion and various analytical methods like quantitative 13C-IS py-GC–MS and multidimensional NMR spectroscopy has been performed to determine the structural and chemical properties of digested biomass.

#### Ruminants microbiota associated depolymerization

Several studies with the rumen microbial community of various ruminants have revealed its ability to degrade plant cell wall to produce saccharides and short chain fatty acids that are eventually absorbed by the host. However, the microbes biocenosis (archaea and bacteria) involved in the biogas production was not effective for lignin barrier disintegration where a considerable fraction of convertible sugars is being untouched. Since the rumen feeds (e.g., forage maize, sorghum and wheat) are also considered as feedstock for bioenergy production, there is a pre-requisite for the exploration of the potential candidate from the rumen microbes for lignocellulosic pretreatment.

Anaerobic fungi, a natural inhabitant of herbivorous gut belonging to the phylum Neocallimastigomycota, decomposes the ingested food [109, 110]. Whereas, LCB pretreatment with these anaerobic fungi favors a better carbohydrate accessibility and the direct utilization of its metabolites in fermentation and methanogenesis. Gut anaerobic fungi has a shorter pretreatment period on comparison with white rot fungi [111]. However, the capacity and ability of rumen fungi involved in the disintegration of forage that has highly lignified walls needs a better understanding. Bootten et al. [112] compared the capabilities of rumen fungi (*Caecomyces communis, Piromyces communis* and *Neocallimastix frontalis*) as mono-culture and co-culturing with *Methanobrevibacter smithii* in the degradation of lignified *Medicago sativa* L. Among them, *Piromyces communis* and *Neocallimastix frontalis* were the efficient degraders showing 37.9% and 33.2% loss of lignin component in *M. sativa*, respectively. Recently, Dollhofer et al. [113] investigated the role of two anaerobic fungal strains (*Neocallimastix frontalis*) in hydrolytic pretreatment of hay biomass that was isolated from the rumen fluid of *Bos taurus taurus* (cow) and *Rupicarpa rupicarpa* (chamois). The pre-processing of LCB with anaerobic fungi have increased the biogas production together with high volatile fatty acid concentrations.

Many scientific reports are available on discovering fungi, protozoan, cellulolytic and hemicellulolytic bacteria from the rumen fluid microbial community. Nevertheless, merely few studies were focused on lignin degradation by rumen associated bacteria as the bacterial detection was hard by means of traditional methods. In one of the earlier studies, *Syntrophococcus sucromutans* an aromatic utilizing rumen fluid bacterium was able to solubilize the wheat cell wall lignin that are labeled with ^14^C isotopes [114]. Baba et al. [115] attempted to evolve a pretreatment strategy by using bacterial flora for methane fermentation of rapeseed. Six taxa of lignin degrading bacteria were identified by next generation sequencing (MiSeq technology) that degrades aromatic compounds like benzenediol, hydroxybenzoate and benzoate that are derived from lignin. Thus, the solubilization of rapeseed with rumen fluid resulted in the enhanced methane production by 1.5 times when compared to untreated biomass. Similarly, rumen fluid pretreatment has effectively enhanced the enzymatic hydrolysis of corn stover, that also resulted in enhanced COD, VFA and reducing sugar production [116].

Taken together, lignin by itself is a complex heterogenous polymer where phenyl propane is linked by ether and carbon–carbon bonds, however the exact chemical structure of lignin and its specific reaction in biodegradation remains unclear.

### Saccharolysis and Ethanol fermentation

**Fig. 5:**
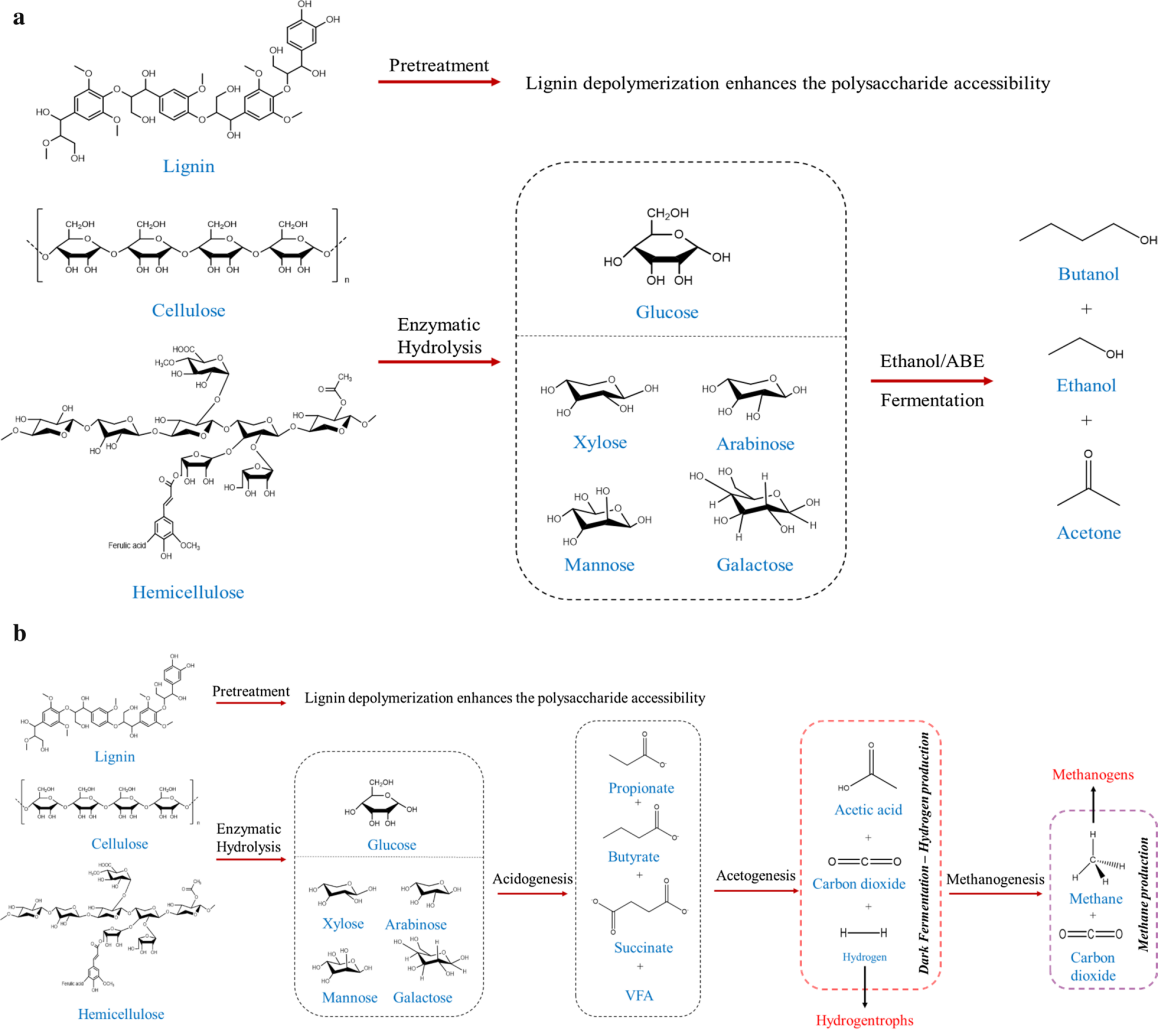
**a** Schematic representation of fermentation strategy adapted for second generation alcohol production. **b** Schematic view of the anaerobic digestion strategy for LCB conversion into biohydrogeand biomethane

Sustainable and renewable holocellulosic polysaccharides (cellulose and hemicellulose) in LCB serves as a potent source of fermentable sugars available for bioethanol production. Saccharolysis of holocellulosic components is the prime stage in the industrial LCB bioconversion into second generation cellulosic ethanol which relics as a fundamental alternative for greenhouse gas emission reduction [117]. However, saccharolysis optimization interferes the cost-cutting of cellulase production as it accounts 40% of the total production cost besides the cheapest renewable carbon of LCB [118]. Recently, exploitation of insects gut microbes/enzymes in the upgradation of saccharolysis to comprehend the polysaccharide chemistry and its industrial claim are gaining interest in renewable fuel and chemicals production. Figure 5a represents the scheme of fermentation strategy for second generation alcohol production.

#### Insects—a foremost microbiome host for cellulosic bioethanol

Insects are one among the novel and robust natural bioreactors colonizing the symbiotic gut microbiome which could act as a biocatalyst to efficiently utilize the LCB as a sole carbon source. Rapid growth rate, ability to utilize a wide spectrum of substrates and high sugar yield are some of the significant characteristic of symbiotic gut bacteria that are in associations with the conventional fungi. Based on the studies, symbiotic gut bacteria isolates from several invertebrates like beetles, worms, snails, caterpillar and termite are more predominantly involved in saccharolysis due to its high cellulolytic activity [61, 119, 120]. In saccharolysis, polysaccharides are breakdown into monosaccharides by the synergistic action of complex multicomponent cellulosome that are preceded by the lignin depolymerization. For example, a facultative and strict anaerobic bacterium (2.5 to 7.4 × 10^8^ bacteria/ mL of hindgut) are isolated from the hindgut larvae of *Pachnoda marginata* (scarab beetle) which has been identified with endoglucanase activity ranging from 0.048–24 U/mL. However, larvae midgut lacks the hemicellulolytic activity, yet the cellulolytic enzymes in midgut act in a pre-cellulolytic phase of intestinal tract which could facilitate the solubilization of LCB. A bacterial strain VPCX2 isolated from *Promicromonospora pachnodae* sp., showed a higher activity of about 20 ± 5 U/mg of xylanase and 24 ± 7 U/mg of CMC-ase using xylose as carbon source. Further, bacterial isolates identified based on 16S rDNA sequencing could ferment glucose and xylose (sole carbon source) into several end products like ethanol, formate, lactate, acetate and succinate [32]. Similarly, an ultramicrobacterium isolated form *Elusimicrobium minutum* produces ethanol as a major end product by fermenting various carbon sources [121]. Szentner et al*.* [122] studied the saccharolytic efficiency of *Zophobas morio* (Superworm) extracts obtained from different developmental stages, where the midgut extracts showed a maximum glucose content (2.480 mg/mL) than the larvae (0.399 mg/mL) when subjected with the microcrystalline cellulose substrate (MCC 20) for 2 h of incubation. The presence of hydrogen bond between the adjacent hydroxyl group affords more stability to crystalline cellulose, whereas amorphous regions of cellulose have only fewer hydrogen linkages between the polymer chains. Based on the studies, its has been reported that the cellulose polymorphism and its morphological properties are the prime concern for saccharolysis. For instance, supermolecular structure of the cellulose could either regulate or affect the efficiency of saccharolysis [122–126].

Integration of saccharolysis and fermentation would be a significant strategy for enhancing the saccharolysis in bioethanol production. In general, simultaneous saccharification and fermentation (SSF) and simultaneous saccharification and co-fermentation (SSCF) of LCB are the most preferable process strategy only when the functions of both biocatalysts are optimal at similar reaction temperature and pH. Thus, the sugar moieties released after saccharolysis are desirably converted into bioethanol by yeast, which improves the end product inhibition caused by sugar accumulation [117]. Enrichment studies has been carried out to isolate cellulose degrading bacteria from various invertebrates. Herein, bacterial candidates are selected based on the maximum cellulose degradation efficiency and future co-cultured with *Saccharomyces cerevisiae* for bioethanol production. For instance, acid pretreated pine biomass inoculated with cellulolytic bacterial isolate 27C64 of *Tipula abdominalis *larvae produced cellulase and xylanase with an activity of about 0.13 IU/mL and 0.19 IU/mL. Further, the bacterial strain has been co-cultured with the *S. cerevisiae *that resulted in 29.8 g/L of ethanol after 48 h of fermentation [127]. A purified endoglucanase of about 43 kDa with 73.210 ± 86 IU specific activity was produced from *Bacillus tequilensis* strain G9 isolates of *Achatina fulica* (snail). Thus, the SSF of enzyme with the yeast strain are found to be efficient in various LCB conversion into bioethanol [75]. A well-known wood feeding natural bioreactor i.e., termite with diverse gut microflora encompass a numerous cellulolytic bacterium namely *Pseudomonas, Acinetobacter,* various species of *Enterobacteriaceae, Bacillaceae* families and *Staphylococcus* being involved in LCB conversion into the fermentable products. An enriched microbial consortium (SSA-6, yeast strain-2 and bacterial strain -4) of *Coptotermes formosanus* was studied to produce various lignocellulolytic enzymes using saw dust under SSF. Among the lignocellulolytic enzymes, cellulase showed a maximum activity of about 15.96 ± 0.87 U mL^−1^ that was relatively higher than ligninolytic (10.77 ± 1.03 U mL^−1^) and xylanolytic (14.65 ± 0.82 U mL^−1^) activity. And further, these SSA-6 derived cocktail enzymes showed a higher ethanol concentration of about 25.5 g/L. The ethanol concentration was 203.6%, 168.4%, and 84.3% higher than individual strains (*Vanrija humicola* SSA-1544, *Starmera dryadoides* SSA-1549^ T^ (yeast) and *Meyerozyma guilliermondii* SSA-1543^ T^ (yeast) [20]. *Cellulomonas* sp., isolates of termite gut microflora are deliberated with cellulase and xylanase hydrolytic efficiency. And the microbial strain is being involved in the of direct conversion of fermentable sugars to secondary metabolites using various agricultural substrate (rice straw, corn stover and cotton stalk). Herein, maximum amount of reducing sugar released was observed to be 18.90 mg/L using corn stover as substrate whereas, direct SSF resulted in 0.425 g/L of ethanol and 0.772 g/L of lactate [128]. On the other hand, Tsegaye et al. [61] investigated the hydrolytic ability of *Bacillus sp.* BMP01 strain of *Cryptotermes brevis* (wood feeding termites) via separate hydrolysis and simultaneous pretreatment and hydrolysis of wheat straw. The result indicates that the total reducing sugar yield after 16 days (439 mg/g) was 9.45% higher in case of separate hydrolysis as compared to the simultaneous treatment (360 mg/g). Currently, xylose fermenting yeast isolates of *Reticulitermes chinensis* are involved in the complex LCB degradation and its bioconversion into simple xylose sugar moieties that could serve as a novel symbiotic microbial candidate for LCB derived carbon recycle into bioethanol synthesis [129]. Similarly, apart from ethanol fermentation, Ali et al. [130] has isolated the 38 novel MnP producing yeast strains from termite gut symbionts. Here, an oleaginous yeast consortium (NYC-1) has been applied for evaluating the combined process of azo dye degradation, lipid accumulation and biodiesel production.

In case of rumen, *C. puniceum* strain Ru6C and *xylanolyticum* Ru15 isolated from cattle ruminal content exhibited high xylanase and endoglucanse activity, respectively. These bacterial strains are utilized as a consortium for the simultaneous production of ethanol and biohydrogen from LCB [131]. Thus, saccharolysis are widely adopted in LCB bioconversion, where innumerable synergistic bacteria, fungi and enzymes of gut microbiome serves as a carbon recycler owing to its degradability, mild operating conditions and absence of formation of toxic inhibitors of subsequent fermentation as compared to chemical means of hydrolysis.

### Hydrogen production

Apart from ethanol production, ruminants and insects (particularly termites) microbial symbionts would serves as the most predominant candidate for hydrogen biosynthesis, which is an intermediate linking the carbohydrate polymer fermentation with reductive acetogenesis and methanogenesis.

#### Bio-mimicking of ruminal symbionts in hydrogen production

Rumen, the first and the foremost chamber in the digestive tract of all animals where the ruminal microflora supplies all the essential nutrients to the small intestine and then the energy produced from the short chain organic acids derives their metabolism [29]. In the anaerobic digestion process of rumen the utilization of molecular hydrogen dominates the methane production [132, 133]. Thus, the rumen fermentation could be manipulated to decrease the methane production and to favor the acetate production pathway which remains as a prime energy source. Acetogenic bacteria would possess the competitiveness for molecular hydrogen and might perform the electron transport phosphorylation in the presence of cytochrome [134]. Thus, in turn enhances the ability of the ruminal symbionts to produce only acetate from H_2_ and CO_2._ Rieu-Lesme et al. [134] attempted to isolate acetogenic bacteria from deer rumen proceeded by evaluating the factors affecting their competitiveness for hydrogen production. In this study, hydrogen, acetate, CO_2_, ethanol, succinate and lactate are the major fermentation end products. Chang et al*.* [29] have established an rumen bacterial consortium which bio-mimics the ruminal microflora for the production of bio-hydrogen (~ 60 mL) and reducing sugar (~ 0.20 mg/mL) using napiergrass as substrate. Based on the study, all the *Clostridial* strains are more predominantly involved in the production of hydrogen from the metabolized sugars. On the other hand, the dominant *Clostridial* strains isolated from rumen by Ho et al. [131] showed higher productivity (*Clostridium puniceum* Ru6 (3379.2 mmol) and *C. xylanolyticum* (1970.6 mmol)) as monoculture on comparison with the bacterial consortium (*C. xylanolyticum, C. papyrosolvens, C. beijerinckii, C. puniceum, C. putrifaciens, and Ruminococcus* sp.,).

During fermentation, the antagonistic effect in the microbial consortium would inhibit the hydrogen yield. Alcohol formation determines the electrons available within NADH which are unfavorable for hydrogen production, yet passive balancing of ethanol and hydrogen production could be feasible. Thus, a numerous bacterial strain discovered so far are being responsible for the carbon recycling through the biomass mineralization that occurs inside the rumen.

#### Termite symbionts based bio-mimicking in hydrogen production

Among insects, termites are predominantly studied in hydrogen bio-mimicking for its high potential and concerning respiratory activity. The daily turnover rate of hydrogen in termite gut was observed to be 9–33 m^3^ of hydrogen per m^3^ of termite hindgut [135]. On comparison, the termite paunch is 10^8^ times smaller than the rumens, which results in high oxygen influx (500 times) per unit volume. The transit time taken for the ingested forages of termite is just one day. Owing to this, bio-mimicking of termite gut microbes via in vitro co-culturing approach would be laid-back. Taken together, the synergistic relationship of termite gut symbionts on hydrogen production was studied by Mathew et al. [136]. Herein, termite gut environment is bio-mimicked by co-culturing the isolates of *Odontotermes formosanus* namely *Bacillus* and *Clostridium* sp., in batch mode using difference carbon source. The study suggests that, the mutualistic act of *Bacillus* sp., created an anaerobic condition required for the *Clostridium* growth which resulted in maximum hydrogen production (4.08 mmol/mL of hydrogen using glucose as substrate).

Some of the previous studies stated that the isolation of bacterial strains (*Clostridium* sp., *Enterobacter cloacae* and *E. aerogenes)* from the termite gut are classified as facultative and obligate hydrogen anaerobes based on its hydrogen producing ability by dark fermentation [137, 138]. Above all, strict anaerobes are proven as a most effective candidate in fermentative hydrogen production. Nevertheless, the growth of obligate anaerobes would be inhibited by low oxygen concentration, which in turn comprehends the certain limits in hydrogen fermentation. On the other hand, even in depleted oxygen conditions, enteric bacteria utilizing the available oxygen can retain the H_2_ producing ability [29]. *Enterobacter cloacae* KBH3 isolate was proven to be a potent hydrogen producer that resulted in a high production rate of about 180.74 mL H_2_/L/h and the hydrogen yield was 1.8 mol H_2_/ mol of glucose under batch fermentation. An increase in cumulative hydrogen production was observed which is contributed by subsequent formate consumption by *E. cloacae* KBH3 in the reaction [139].

In order to understand the competitive mechanism of gut acetogen for in situ hydrogen production, an attempt has been made to isolate prominent bacteria such as *Sporoniusa termitida* and *Acetonema Iongum* from wood feeding termites namely *Nasutiterines nigriceps* and *Pterotermes occidentis* respectively. Ramachandran et al. [140] investigated the fermentative pattern of *Clostridium termitidis* strain CT1112, isolated from *Nasutitermes lujae* for hydrogen production under batch cultivation. The fermentative end products such as ethanol, CO_2_, acetate, lactate and formate were obtained with α-cellulose and cellobiose as sole carbon sources. Being an obligate anaerobe, it showed a maximum yield of about 4.6 mmol/L and 7.7 mmol/L of hydrogen for cellobiose and α-cellulose, respectively. Thus, the termite nutrition depends on bacterial acetogenesis for acetate oxidation in order to meet the respiratory requirements [141]. Though, the bacterial isolates of fungus cultivating and soil feeding termites possess less acetogenesis rate and high methane emission rate than the wood feeding counterparts. An attempt has been made by Kane and Breznak, [142] to discover acetogenic bacteria analog (*Clostridium mayombei* sp.) from the soil feeding termites (*Cubitermes speciosus*) where the bacterial acetogenesis was not presiding in hindgut fermentation. The maximum hydrogen produced from strict anaerobe was around 13.4 mmol/100 mmol of glucose with 85% of carbon recovery. Globally, several ongoing studies are focused on fermentative optimization for H_2_ production from gut symbionts in order to augment its net energy yield and production rates.

### Anaerobic digestion

Microbial imbalance is the prime factor that influences on relative amount of biogas produced during anaerobic digestion. A notable number of biogas digesters have inability to operate efficiently and it would take weeks to months for biogas production from the plant fibers. However, the gastrointestinal tract of herbivores like insects and ruminants have merely two days as residence time for the bioconversion of plant fiber into methane and VFA [143, 144]. Idealistic evolutionary establishes an efficient gut/rumen symbiotic microbial community of the herbivores which aids in the LCB digestion and thus a detailed insight into the symbiotic microbial role has gained a great interest [145].

#### Role of ruminants microflora in bioaugmented digesters

Ruminants, a finest example for the symbiotic relation of microbes where protozoa, bacteria, anaerobic fungi and archaea work synergistically to produce VFA and methane by LCB degradation. Based on the previous studies on exploiting the rumen fluid microbes ability in LCB conversion, the symbiotic ruminal microbes are observed with high cellulolytic activity rather than any other anaerobic digester inoculum [146]. Bioaugmentation of specific microbes with native digesters would be a promising enrichment strategy for anaerobic digestion. Recently, bioaugmentation studies on improving the hydrolysis rate has gained interest in various plant biomass namely corn straw [147], wheat straw [148], cattail [149] and corn processing waste [150] for the enhanced methane production. Ozbayram et al. [151] investigated the bioaugmentation potential of enriched sheep rumen cellulolytic microbes in methane production. The enriched microbial isolates resulted in an average yield of 154 mL/g Volatile Solids (VS) using wheat straw as a substrate over a period of 30 days in a controlled batch reactor. Influence of cattle gender on anaerobic digestion of wheat straw along with the selection of more efficient bacteria from heifers and bulls (24 months old) ruminal fluid was studied based on its nutrient digestibility and distinctive rumen fermentation [152]. Results suggest that bulls ruminal microbes serve as a most predominant methane producer with a concentration of about 288.2 mL/g of VS that was 35.2% higher than the heifers rumen fluid. Similarly, Bittante et al. observed that the bulls have high nutrient digestibility and growth performance than heifers and thus the application of its ruminal microbes in anaerobic digestion would provide an efficient biogas and VFA production [153]. Meanwhile, study on rumen fermentation by in vitro batch culture has been increased, yet the diet, rumen processing method and the sampling time of donor rumen contents remains as a foremost regulating factor. Subsequently, Mateos et al. [154] studied the outcome of three diverse rumen fluid processing technique based on rumen microbiota population and its effect on in vitro anaerobic fermentation parameters. In this study, four sheep with fistulae rumen was fed with alfalfa hay and concentrate diet (2:1), later the rumen content were subjected to the processing methods like SQ (Squeezed by four layer of cheese cloth); FL (SQ + nylon cloth (100 µm) filtration) and STO (Stomacher® blending + SQ). Among them, in vitro fermentation using STO resulted in higher methane production (510 µmol) within 24 h of incubation as compared to other methods. Nevertheless, the hydrolysis of highly lignified LCB perhaps remains as a rate limiting step in anaerobic digestion. Hence, the microwave irradiation pretreatment of LCB prior to the fermentation has improved the anaerobic digestibility of rumen microbes in cattail feedstock conversion, where the product formation rate and yield were 32% and 19% higher than the conventional anaerobic digestion [155]. Similarly, date leaf and wheat straw substrates has been subjected to the microbial pretreatment by ligninolytic bacteria isolated from termite gut symbionts and further these pretreated by-products has been utilized as substrate for in vitro anaerobic fermentation [156].

#### Role of insects microflora in bioaugmented digesters

In parallel to ruminants, insects like scarab beetles and termites are also familiar in methane production, where methanogenesis originates in their hindgut [157]. Scarab beetle larva have distinctly enlarged midgut and hindgut which can digest the ingested plant fibers up to 65% and thus, the gut microbes are the dynamic considerate of anaerobic digestion. In beetle, midgut unveils a pre-cellulolytic phase environment at highly alkaline pH (11–12), whereas, the hindgut harbors methanogens together with a vast number of other bacteria (10^10^–10^11^ mL/gut) [121]. Herein, the midgut fermentation is being coupled with the hindgut methanogenesis via the formate transportation in hemolymph. For instance, Cazemier et al. [158] compared the efficiency of hindgut microbial consortia of *Pachnoda marginata* larvae with rumen fluid cultures in plant fibers degradation, methane and VFA production under batch fermentation. The result indicated that the methane and VFA (acetate, butyrate and propionate) production was high in rumen fluids as compared to hindgut consortia. However, in case of rumen, the microflora activity has a constrained narrow temperature range (35–40 ºC).

Culture independent characterization of gut microbial community of humus feeding larva revealed that the bacterial consortium (archaea and bacteria) identified with fermentative metabolism are phylogenetically dominant. In addition, the ability of hindgut microbiota in methane production under aerobic conditions are deliberated [159]. Similarly, Egert et al. [160] discovered the pattern of microbial consortia from *Melolontha melolontha* larvae are analogous to the former larva consortia with the foremost variance in methanogens. Thus, the substantial similarity has been found in the insects gut consortia from Coleoptera: Scarabaeida (Order: Subfamily).

Cellulose digestion and methane producing ability of termites are well documented in several studies. In termite gut, P3/4a compartment has the pronounced emission of methane as compared to P1 and P4b which indicates that the methane production is influenced by the diverse microbes colonized in distinct gut compartments [157, 161, 162]. Termites, being the microscale lignocellulolytic bioreactor, where the gut symbionts produce some fermentative products such as propionate, acetate and other carboxylates (volatile fatty acids) from the LCB degradation regardless of the termite species. Over the evolution of 150 million years, various strategies have been developed by termites symbiotic microbiome in converting the LCB into value added products (hydrogen, methane or volatile fatty acids) [163–165]. Though, most of the studies are focused on the isolation, identification and characterization of termite gut microbes, some studies have also elucidated the detailed enzymes production profile with advanced metagenomic approaches [165]. However, only limited studies are being focused on exploring the termite gut microbiota as an inoculum for the production of industrially relevant VFA [165]. More often, a mixed anaerobic bacterial community are explored to produce various carboxylate like propionic, butyric and acetic acid under non-sterile conditions [166]. Auer et al. [31] developed a feasible technology in carboxylate production by stabilizing the gut microbial community isolated from the four higher termites species namely, *Termes hospes*, *Nasutitermes ephratae, Microcerotermes parvus,* and *Nasutitermes lujae* (undescribed-closely related species). Later, Lazuka et al. [167] enriched and stabilized the gut micro symbionts of *Nasutitermes ephratae* for valuable carboxylates production from wheat straw under anaerobic condition. The overall schematic outline of anaerobic digestion for LCB conversion into biohydrogen and biomethane has been depicted in Fig. 5b.

## Challenges and future perspectives

A paradigm shift has been attained towards harnessing the carbon rich second generation LCB for biofuels and value-added chemicals production. Yet, an array of challenges related to logistics, deployment of energy efficient LCB conversion strategy and its technoeconomic impact evaluation are still existing. These issues could be resolved by uncovering the potential microbial/enzyme candidate with desired characteristic that act as a natural carbon recycler, thereby generating the sustainability and economic feasibility. To strengthen the perspective of second generation biofuels industry, implementation of a centralized markets is required to provide homogenous supply routes and integrated bioprocess strategy for the cost competitive second generation biofuels. Further, to hit the market demand, the choice of second-generation technology relies mainly on a consolidated bioconversion strategy that employs the synergistic gut microsymbionts along with the government biofuel policy and subsidiaries. Thus, providing a “cradle to grave approach” with the recycling framework of growing waste production.

## Conclusion

Recently, low cost and highly efficient LCB based biorefinery strategy for the biofuels and renewable chemicals production has been vastly explored. Based on the studies, several xylophagous insects and herbivores animals have been discovered as a potent source for LCB bioconversion owing to its synergistic symbionts. Advent of various culture independent metagenomic, meta-transcriptomic and in situ stable isotope based probing techniques for uncultivable symbionts aids in the identification of novel CAZymes (β-1,4 glucanase and lytic polysaccharide monooxygenase), and exploration of its functional and metabolic characteristics. Many recent reports have highlighted the significance of gut/rumen microbial community enrichment and compares the efficiency of enriched microbial consortia to establish a consolidated biorefinery framework for second generation LCB. Further, mining of LCB degrading genes encoding for various lignocellulolytic enzymes production has provoked the potential application of symbiotic microflora in bio-mimicking. Hence, a single ruminant or an insect species could feasibly afford all the vital biocatalyst that are desired for LCB based biorefinery.

## Data Availability

The figures and the sources of information provided in the manuscript will be provided based on the request.
